# Crystal structure of a new spiro-polytetra­hydro­furan compound with translational pseudosymmetry: *rac*-(2*S*,2′*S*,5′*R*)-2-methyl-5′-[(1*R*,2*R*,5*S*,5′*R*)-1,4,4,5′-tetra­methyl­dihydro-3′*H*-3,8-dioxa­spiro[bi­cyclo­[3.2.1]octane-2,2′-furan]-5′-yl]hexa­hydro[2,2′-bi­furan]-5(2*H*)-one

**DOI:** 10.1107/S2056989017006065

**Published:** 2017-04-28

**Authors:** Vincenzo Piccialli, Angela Tuzi, Roberto Centore

**Affiliations:** a Dipartimento di Scienze Chimiche, Università degli Studi di Napoli ‘Federico II’, Complesso di Monte S. Angelo, Via Cinthia, 80126 Napoli, Italy

**Keywords:** crystal structure, pseudosymmetry, pseudotranslation, poly-THF compounds, spiro-compounds

## Abstract

The title compound, C_22_H_34_O_6_, is a product of oxidation of squalene with the catalytic system RuO_4_(cat.)/NaIO_4_. The asymmetric unit contains two crystallographically independent mol­ecules of very similar geometry approximately related by the non-crystallographic translation vector **c**/2. As a consequence, the average diffracted intensity in the *hkl* layers with odd *l* is systematically lower than in the layers with even *l*. In one mol­ecule, the lactone ring and part of the adjacent tetra­hydro­furan ring are disordered over two orientations with refined occupancy ratio of 0.831 (10):0.169 (10). The crystal structure is mainly governed by van der Waals forces.

## Abstract 2

The title compound crystallizes in the *P* 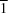 space group, with two crystallographically independent mol­ecules approximately related by the non-crystallographic translation vector **c**/2.

## Chemical context   

**Figure 1:**
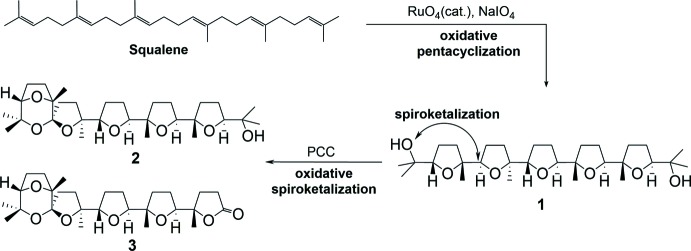
Scheme of the synthesis, showing the formation of polytetra­hydro­furan compounds by oxidative cyclization of squalene with RuO_4_ and the formation of spiro-polytetra­hydro­furan compounds by subsequent oxidative spiro­ketalization with pyridinium chloro­chromate (PCC).

Our group has long been involved in the synthesis of new biologically active heterocyclic compounds (D’Errico *et al.*, 2011 ▸, 2012*a* ▸,*b* ▸; Oliviero *et al.*, 2008 ▸, 2010*a* ▸,*b* ▸; Centore *et al.*, 2013 ▸; Iovine *et al.*, 2014 ▸). In particular, we have developed a number of new catalytic oxidative processes mediated by transition metal oxo-species (Piccialli *et al.*, 2009 ▸, 2013 ▸) leading to the stereoselective formation of mono- and poly-tetra­hydro­furan (THF) compounds (Piccialli, 2014 ▸), as well as spiro­ketal compounds. THF-containing substances are widely distributed in nature and display a broad range of biological activities such as cation transport, citotoxic, pesticidal, anti-tumor and immunosuppressive activity. The oxidation of squalene with catalytic amounts of RuO_4_ (Bifulco *et al.*, 2003 ▸; Piccialli *et al.*, 2007 ▸) is particularly impressive since it undergoes a stereoselective cascade process leading to the penta-THF compound **1** (Fig. 1 ▸) in a straightforward way and high yields (50% for five consecutive cyclization steps; 87% per cyclization step). In this way, multi-gram amounts of this substance can be easily obtained starting from a cheap parent material. Compound **1**, in turn, has been used as the starting material for the synthesis of a number of new poly-THF and spiro­ketal substances such as, *inter alia*, compounds **2** and **3** (Fig. 1 ▸) that have shown anti-cancer activity against ovarian (HEY) and breast cancer-derived (BT474) cell lines (Piccialli *et al.*, 2009 ▸).

Based on the known reactivity of RuO_4_ (Piccialli *et al.*, 2008 ▸, 2010 ▸), we anti­cipated that truncated spiro­compounds structurally related to **2** and **3** of Fig. 1 ▸ could likely be produced just during the oxidation of squalene with RuO_4_. We report here that a search for this type of products for biological assays and SAR studies resulted in the isolation of the title compound, a substance possessing the same tricyclic spiro­ketal terminal moiety found in **2** and **3** and strictly related to them. Although extensive NMR studies allowed to determine the structure of this compound, the configuration of some chiral centres could not be unambiguously determined. This prompted us to undertake the X-ray diffraction study of this compound.
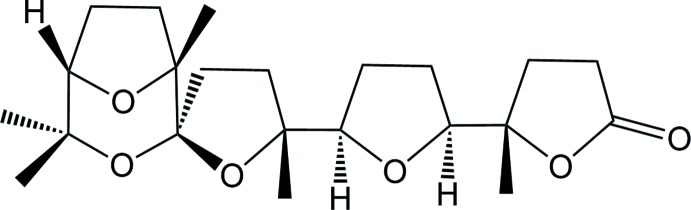


## Structural commentary   

**Figure 2:**
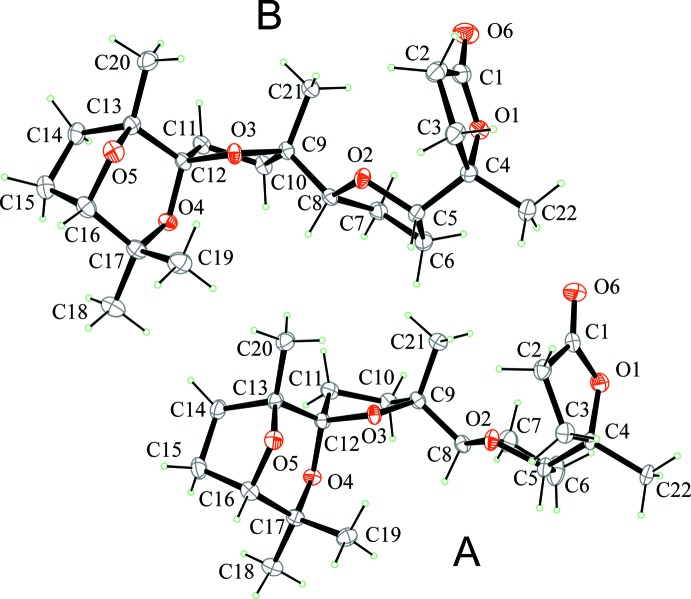
View of the mol­ecular structures of the title compound. Displacement ellipsoids are drawn at the 30% probability level. Only the major component of the disordered lactone ring of mol­ecule A is shown for clarity.

**Figure 3:**
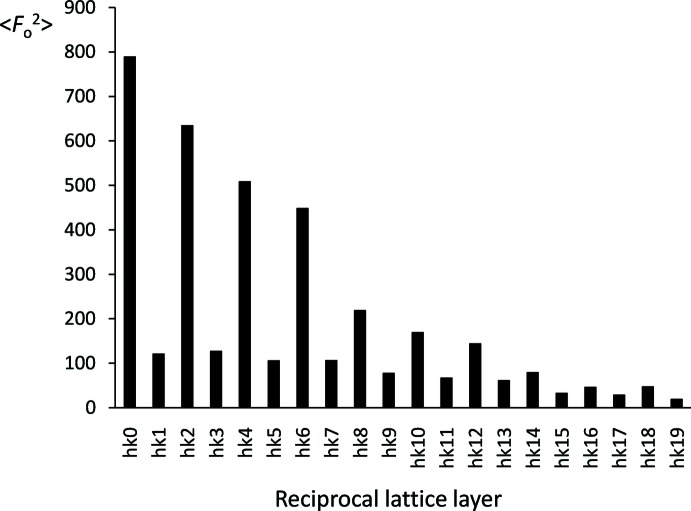
Average squared observed structure factor per reciprocal lattice layer, as a function of the *l* index.

The asymmetric unit contains two mol­ecules of very similar conformation, shown in Fig. 2 ▸. The two mol­ecules are approximately related by a translation vector that can be determined by calculating the difference between the homologue coordinates of corresponding atoms in the two mol­ecules *A* and *B*. In this way, fairly constant values of the differences are obtained that, averaged over all the couples of (non H) corresponding atoms in the two mol­ecules, give the final values: <Δ*x*>= −0.02 (3), <Δ*y*>= 0.01 (16) and <Δ*z*>= 0.50 (2). This means that the two mol­ecules, on average, are related by the translation vector **t** = **c**/2. This pseudosymmetry has consequences on the diffraction pattern. Of course, if the symmetry were truly crystallographic, then all reflections *hkl* with *l* odd would have null intensity, because each structure factor *F_hkl_* would bear a factor (1 + *e^i^* ^π*l*^). The structure could be described in a cell of half the volume and *Z′* = 1. This is not the case, because the translational symmetry is not crystallographic. However, a trace of it can be found in the fact that the average diffracted intensity in the *hkl* layers with odd *l* is systematically lower than in the layers with even *l*. This is shown in the histogram of Fig. 3 ▸, in which we have averaged the measured *F*_o_^2^ over each layer. The modulation of the average diffracted intensity between layers with even and odd *l* is dramatically evident.

**Figure 4:**
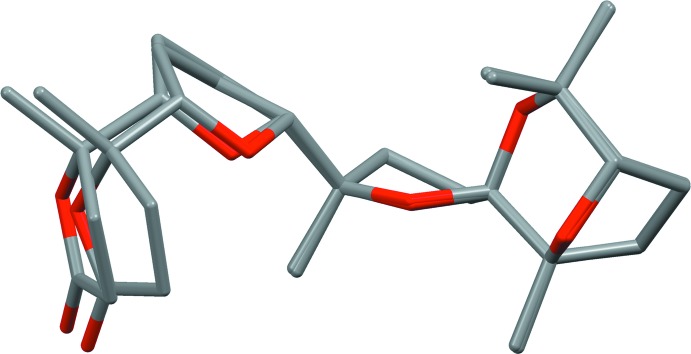
Overlay of the two independent mol­ecules *A* and *B*. For mol­ecule *A*, only the major component of the disordered lactone ring is shown.

The conformation of the two independent mol­ecules is almost the same, with exception for the lactone ring, whose orientation is slightly different (Fig. 4 ▸). In both mol­ecules the five-membered rings O1/C1–C4 and O3/C9–C12 exhibit a twist conformation, while the O2/C5–C8 rings display an envelope conformation with atom C8 at the flap. From the analysis of the mol­ecular structure, it turns out that the relative configuration of the two chiral carbons C8 and C9 in the title compound is inverted as compared with the isomeric compound already reported in literature (compound **10** of Scheme 3 in Piccialli *et al.*, 2009 ▸). Moreover, the title compound shares the relative configuration of all of its seven chiral centres with the corresponding moiety in a *meso*-bis-spiro-compound previously obtained by oxidation of squalene under the same conditions (compound **8** of Scheme 2 in Piccialli *et al.*, 2010 ▸).

## Supra­molecular features   

**Figure 5:**
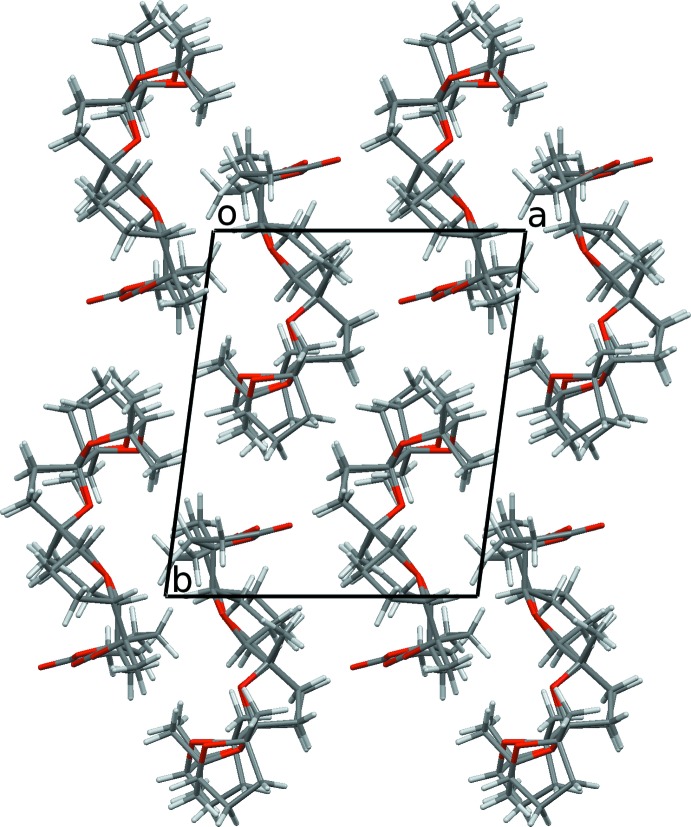
The crystal packing viewed down the *c* axis. For mol­ecule *A*, only the major component of the disordered lactone ring is shown.

The crystal packing is shown in Fig. 5 ▸. Although some intra- and inter­mol­ecular C–H⋯O hydrogen contacts are observed (Table 1 ▸), no classical hydrogen bonds are found and mol­ecules in the crystal are held basically through van der Waals contacts between H atoms.

**Figure 6:**
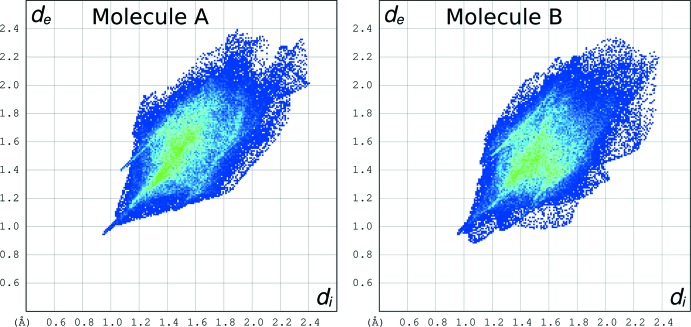
Hirshfeld fingerprint plots of the two crystallographically independent mol­ecules of the title compound.

In order to assess possible packing differences involving the two independent mol­ecules we have examined their Hirshfeld surfaces (Spackman & McKinnon, 2002 ▸; Wolff *et al.*, 2012 ▸). In Fig. 6 ▸ are shown Hirshfeld fingerprint plots of the two independent mol­ecules, while Table 2 ▸ gives relevant mol­ecular parameters.

In the plots, for each point of the Hirshfeld surface enveloping the mol­ecule in the crystal, the distance *d*_i_ to the nearest atom inside the surface and the distance *d*_e_ to the nearest atom outside the surface are reported. The color of each point in the plot is related to the abundance of that inter­action, from blue (low) to green (high) to red (very high).

A common feature of each plot of Fig. 6 ▸ is represented by the central green area around *d*_i_ + *d*_e_ = 3.0 Å, that corresponds to the loose van der Waals contacts present in the packing, and mainly involving H atoms. Another common feature is the sting along the diagonal, down to *d*_i_ = *d*_e_ = 0.9 Å, which reflects points on the Hirshfeld surface that involve nearly head-to-head close H⋯H contacts. This feature is clearly more pronounced in the plot of mol­ecule *A*.

## Database survey   

A search of the Cambridge Structural Database (CSD version 5.38, last update February 2017; Groom *et al.*, 2016 ▸) gave no match for the title compound. A search for spiro-THF compounds gave six hits (GUHXOX, GUHXUD, MUZTEH, MUZTIL, MUZTOR and MUZTUX) all coming from our research group (Piccialli *et al.*, 2009 ▸, 2010 ▸). A search for poly-THF compounds in which one terminal THF group, at least, is in the oxidized lactone form gave three hits: DOJSIE (Still & Romero, 1986 ▸), FAZJEV (Russell *et al.*, 1987 ▸) and GUHXOX (Piccialli *et al.*, 2009 ▸). Finally, the maximum number of consecutive THF units in a poly-THF compound deposited in the CSD is five: ACUWIG (Yang *et al.*, 2012 ▸) and LOJLUR (Xiong & Corey, 2000 ▸).

## Synthesis and crystallization   

The title compound was obtained by oxidation of squalene with RuO_4_(cat.)/NaIO_4_, as previously described (Piccialli *et al.*, 2010 ▸). The crude product was purified by repeated silica-gel column chromatography, eluting with increasing amounts of Et_2_O in hexane. The fractions enriched in the title compound were collected and evaporated under reduced pressure. Further separation by reversed-phase HPLC (Hibar RP-18 columns, 250 × 10 and 250 × 4 mm, eluent MeOH/H_2_O, 6:4 *v*/*v*) gave the pure title compound as an oil. It was dissolved in the minimal amount of MeOH and the solution was left to evaporate slowly overnight at room temperature to give crystals suitable for X-ray diffraction analysis.

## Refinement   

Crystal data, data collection and structure refinement details are summarized in Table 3 ▸. The H atoms were generated stereochemically and were refined using the riding model, with C—H = 0.98–1.00 Å, and with *U*_iso_ = 1.2*U*_eq_(C) or 1.5*U*_eq_(C) for methyl H atoms. A rotating model was used for the methyl groups. The lactone ring and, in part, the adjacent tetra­hydro­furan ring of the independent mol­ecule *A* are disordered over two orientations. The two split positions were refined by applying SADI restraints on bond lengths and SIMU/EADP restraints on thermal parameters. Constraints were also applied to the C4*AA-*–O1*AA* [1.40 (2) Å], C1*AA*—C2*AA* [1.48 (2) Å], C2*AA-*–C3*AA* [1.52 (2) Å] and C3*AA*—C4*AA* [1.54 (2) Å] bond lengths. The final refined occupancy factors of the two components of disorder are 0.831 (10) and 0.169 (10).

## Supplementary Material

Crystal structure: contains datablock(s) global, I. DOI: 10.1107/S2056989017006065/rz5214sup1.cif

Structure factors: contains datablock(s) I. DOI: 10.1107/S2056989017006065/rz5214Isup2.hkl

Supporting information file. DOI: 10.1107/S2056989017006065/rz5214Isup3.cml

CCDC reference: 1545387

Additional supporting information:  crystallographic information; 3D view; checkCIF report

## Figures and Tables

**Table 1:** Hydrogen-bond geometry (Å, °)

*D*—H⋯*A*	*D*—H	H⋯*A*	*D*⋯*A*	*D*—H⋯*A*
C19*A*—H19*A*⋯O3*A*	0.98	2.39	3.041 (3)	124
C19*B*—H19*D*⋯O3*B*	0.98	2.46	3.038 (3)	117
C7*A*—H7*A*1⋯O6*B* ^i^	0.99	2.55	3.464 (4)	154

Symmetry code: (i) 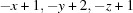.

**Table 2:** Parameters of the Hirshfeld surface of the two crystallographically independent mol­ecules Hirshfeld surface analysis was performed using the program *CrystalExplorer* (Wolff *et al.* 2012 ▸).

Mol­ecule	volume (Å^3^)	area (Å^2^)	globularity	asphericity
*A*	506.20	398.92	0.770	0.127
*B*	500.14	401.64	0.759	0.151

**Table 3:** Experimental details

Crystal data	
Chemical formula	C_22_H_34_O_6_
*M*_r_	394.49
Crystal system, space group	Triclinic, *P* 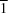
Temperature (K)	173
*a*, *b*, *c* (Å)	11.750 (4), 13.805 (1), 14.737 (2)
α, β, γ (°)	68.622 (11), 67.780 (19), 88.557 (15)
*V* (Å^3^)	2043.0 (9)
*Z*	4
Radiation type	Mo *K*α
μ (mm^−1^)	0.09
Crystal size (mm)	0.50 × 0.50 × 0.12

Data collection	
Diffractometer	Bruker–Nonius KappaCCD
Absorption correction	Multi-scan (*SADABS*; Bruker, 2001 ▸)
*T*_min_, *T*_max_	0.945, 0.973
No. of measured, independent and observed [*I* > 2σ(*I*)] reflections	30708, 9260, 5634
*R*_int_	0.053
(sin θ/λ)_max_ (Å^−1^)	0.650

Refinement	
*R*[*F* ^2^ > 2σ(*F* ^2^)], *wR*(*F* ^2^), *S*	0.052, 0.123, 1.05
No. of reflections	9260
No. of parameters	596
No. of restraints	62
H-atom treatment	H-atom parameters constrained
Δρ_max_, Δρ_min_ (e Å^−3^)	0.35, −0.21

Computer programs: *COLLECT* (Nonius, 1999 ▸), *DIRAX/LSQ* (Duisenberg *et al.*, 2000 ▸), *EVALCCD* (Duisenberg *et al.*, 2003 ▸), *SIR97* (Altomare *et al.*, 1999 ▸), *SHELXL2016* (Sheldrick, 2015 ▸), *ORTEP-3 for Windows* and *WinGX* (Farrugia, 2012 ▸) and *Mercury* (Macrae *et al.*, 2006 ▸).

## supplementary crystallographic information

### Crystal data

**Table:**

C_22_H_34_O_6_	*Z* = 4
*M**_r_* = 394.49	*F*(000) = 856
Triclinic, *P*1	*D*_x_ = 1.283 Mg m^−^^3^
*a* = 11.750 (4) Å	Mo *K*α radiation, λ = 0.71073 Å
*b* = 13.805 (1) Å	Cell parameters from 170 reflections
*c* = 14.737 (2) Å	θ = 3.8–23.6°
α = 68.622 (11)°	µ = 0.09 mm^−^^1^
β = 67.780 (19)°	*T* = 173 K
γ = 88.557 (15)°	Prism, colourless
*V* = 2043.0 (9) Å^3^	0.50 × 0.50 × 0.12 mm

### Data collection

**Table:**

Bruker–Nonius KappaCCD diffractometer	9260 independent reflections
Radiation source: normal-focus sealed tube	5634 reflections with *I* > 2σ(*I*)
Graphite monochromator	*R*_int_ = 0.053
Detector resolution: 9 pixels mm^-1^	θ_max_ = 27.5°, θ_min_ = 2.9°
CCD rotation images, thick slices scans	*h* = −15→14
Absorption correction: multi-scan (*SADABS*; Bruker, 2001)	*k* = −17→17
*T*_min_ = 0.945, *T*_max_ = 0.973	*l* = −19→17
30708 measured reflections	

### Refinement

**Table:**

Refinement on *F*^2^	Primary atom site location: structure-invariant direct methods
Least-squares matrix: full	Secondary atom site location: difference Fourier map
*R*[*F*^2^ > 2σ(*F*^2^)] = 0.052	Hydrogen site location: inferred from neighbouring sites
*wR*(*F*^2^) = 0.123	H-atom parameters constrained
*S* = 1.05	*w* = 1/[σ^2^(*F*_o_^2^) + (0.0357*P*)^2^ + 1.0216*P*] where *P* = (*F*_o_^2^ + 2*F*_c_^2^)/3
9260 reflections	(Δ/σ)_max_ < 0.001
596 parameters	Δρ_max_ = 0.35 e Å^−^^3^
62 restraints	Δρ_min_ = −0.21 e Å^−^^3^

### Special details

**Table:**

Geometry. All esds (except the esd in the dihedral angle between two l.s. planes) are estimated using the full covariance matrix. The cell esds are taken into account individually in the estimation of esds in distances, angles and torsion angles; correlations between esds in cell parameters are only used when they are defined by crystal symmetry. An approximate (isotropic) treatment of cell esds is used for estimating esds involving l.s. planes.
Refinement. Reflection 1 1 0 was not considered in the refinement, because its intensity was affected by the beamstop. The lactone ring and, in part, the adjacent tetrahydrofuran ring of the independent molecule A are disordered over two sites. The two split positions were refined by using some restraints on bond lengths and thermal parameters.

### Fractional atomic coordinates and isotropic or equivalent isotropic displacement parameters (Å^2^)

**Table:**

	*x*	*y*	*z*	*U*_iso_*/*U*_eq_	Occ. (<1)
O1A	0.7941 (4)	1.1654 (4)	0.1721 (4)	0.0375 (9)	0.831 (10)
O6A	0.7350 (4)	1.1861 (3)	0.3254 (3)	0.0629 (11)	0.831 (10)
C1A	0.8077 (5)	1.1558 (3)	0.2620 (3)	0.0366 (11)	0.831 (10)
C2A	0.9212 (4)	1.1059 (3)	0.2652 (3)	0.0326 (9)	0.831 (10)
H2A1	0.898512	1.033649	0.319797	0.039*	0.831 (10)
H2A2	0.973206	1.147172	0.281244	0.039*	0.831 (10)
C3A	0.9891 (4)	1.1049 (4)	0.1557 (3)	0.0319 (9)	0.831 (10)
H3A1	1.021556	1.037141	0.159969	0.038*	0.831 (10)
H3A2	1.059075	1.162374	0.112328	0.038*	0.831 (10)
C4A	0.8909 (5)	1.1204 (4)	0.1084 (4)	0.0259 (11)	0.831 (10)
C5A	0.8346 (6)	1.0140 (5)	0.1266 (7)	0.0297 (10)	0.831 (10)
H5A	0.901092	0.985368	0.081019	0.036*	0.831 (10)
C22A	0.9373 (7)	1.1937 (5)	−0.0082 (4)	0.0398 (14)	0.831 (10)
H22D	1.002179	1.163839	−0.051118	0.060*	0.831 (10)
H22E	0.868319	1.202101	−0.031208	0.060*	0.831 (10)
H22F	0.971293	1.262267	−0.017197	0.060*	0.831 (10)
C6A	0.7190 (5)	1.0057 (4)	0.1019 (4)	0.0549 (15)	0.831 (10)
H6A1	0.690542	1.075172	0.078964	0.066*	0.831 (10)
H6A2	0.738223	0.978332	0.044950	0.066*	0.831 (10)
C7A	0.6212 (2)	0.93056 (19)	0.20438 (18)	0.0418 (6)	0.831 (10)
H7A1	0.571273	0.884768	0.190982	0.050*	0.831 (10)
H7A2	0.565324	0.968705	0.244885	0.050*	0.831 (10)
O1AA	0.756 (2)	1.1546 (19)	0.167 (2)	0.041 (4)	0.169 (10)
O6AA	0.669 (3)	1.1829 (15)	0.3139 (15)	0.086 (7)	0.169 (10)
C1AA	0.756 (3)	1.1610 (19)	0.257 (2)	0.047 (5)	0.169 (10)
C2AA	0.874 (2)	1.1313 (18)	0.2664 (14)	0.035 (4)	0.169 (10)
H2A3	0.859514	1.062948	0.325806	0.042*	0.169 (10)
H2A4	0.913343	1.185033	0.278771	0.042*	0.169 (10)
C3AA	0.953 (2)	1.124 (2)	0.1630 (19)	0.051 (6)	0.169 (10)
H3A3	1.002862	1.065073	0.173371	0.061*	0.169 (10)
H3A4	1.009976	1.190119	0.113873	0.061*	0.169 (10)
C4AA	0.858 (2)	1.106 (2)	0.122 (2)	0.029 (4)	0.169 (10)
C5AA	0.819 (3)	1.005 (3)	0.118 (4)	0.029 (4)	0.169 (10)
H5AA	0.873705	0.982478	0.060252	0.034*	0.169 (10)
C22C	0.908 (4)	1.194 (3)	0.013 (2)	0.052 (7)	0.169 (10)
H22G	0.982037	1.176475	−0.034170	0.078*	0.169 (10)
H22H	0.844443	1.204721	−0.017063	0.078*	0.169 (10)
H22I	0.928603	1.259043	0.019373	0.078*	0.169 (10)
C6AA	0.690 (2)	1.0325 (14)	0.1218 (19)	0.044 (5)	0.169 (10)
H6A3	0.666426	1.091612	0.144946	0.052*	0.169 (10)
H6A4	0.682395	1.046955	0.053668	0.052*	0.169 (10)
C7AA	0.6212 (2)	0.93056 (19)	0.20438 (18)	0.0418 (6)	0.169 (10)
H7A3	0.595604	0.889371	0.171006	0.050*	0.169 (10)
H7A4	0.545201	0.942885	0.256140	0.050*	0.169 (10)
C8A	0.69801 (17)	0.86778 (15)	0.26248 (15)	0.0272 (4)	
H8A	0.729499	0.812376	0.234032	0.033*	
C9A	0.63505 (17)	0.81626 (15)	0.38235 (16)	0.0267 (4)	
C10A	0.52124 (18)	0.73779 (16)	0.41873 (17)	0.0318 (5)	
H10C	0.443582	0.769365	0.438859	0.038*	
H10D	0.526499	0.712579	0.362577	0.038*	
C11A	0.52838 (18)	0.64930 (16)	0.51468 (16)	0.0317 (5)	
H11A	0.494122	0.665891	0.579086	0.038*	
H11B	0.483960	0.582090	0.528605	0.038*	
C12A	0.66756 (17)	0.64591 (15)	0.47904 (15)	0.0255 (4)	
C13A	0.71980 (18)	0.60110 (15)	0.56538 (15)	0.0286 (4)	
C14A	0.6708 (2)	0.48395 (16)	0.63024 (16)	0.0357 (5)	
H14C	0.662567	0.463781	0.704377	0.043*	
H14D	0.589222	0.467051	0.630610	0.043*	
C15A	0.7696 (2)	0.42793 (16)	0.57253 (17)	0.0378 (5)	
H15C	0.813672	0.388297	0.617102	0.045*	
H15D	0.732000	0.379106	0.553233	0.045*	
C16A	0.85690 (19)	0.51736 (16)	0.47390 (17)	0.0315 (5)	
H16A	0.942948	0.498125	0.451297	0.038*	
C17A	0.81794 (17)	0.55502 (16)	0.38037 (16)	0.0277 (4)	
C18A	0.8093 (2)	0.46739 (17)	0.34289 (18)	0.0366 (5)	
H18A	0.790395	0.495166	0.279531	0.055*	
H18B	0.743498	0.411521	0.399243	0.055*	
H18C	0.888624	0.439043	0.325743	0.055*	
C19A	0.90815 (18)	0.64781 (17)	0.28822 (16)	0.0348 (5)	
H19A	0.906290	0.707245	0.309888	0.052*	
H19B	0.884042	0.667780	0.227673	0.052*	
H19C	0.992157	0.628105	0.267823	0.052*	
C20A	0.7055 (2)	0.66729 (17)	0.63017 (17)	0.0385 (5)	
H20A	0.747140	0.738635	0.583015	0.058*	
H20B	0.742931	0.636565	0.681808	0.058*	
H20C	0.617240	0.669606	0.667730	0.058*	
C21A	0.6049 (2)	0.89513 (17)	0.43579 (17)	0.0365 (5)	
H21D	0.682061	0.933573	0.422843	0.055*	
H21E	0.558584	0.857879	0.512363	0.055*	
H21F	0.554531	0.944625	0.406515	0.055*	
O2A	0.80145 (12)	0.94254 (11)	0.23244 (10)	0.0301 (3)	
O3A	0.72123 (11)	0.75191 (10)	0.41868 (10)	0.0261 (3)	
O4A	0.69431 (11)	0.58450 (10)	0.41502 (10)	0.0265 (3)	
O5A	0.85203 (12)	0.60137 (11)	0.51048 (11)	0.0306 (3)	
C1B	0.7270 (2)	1.17216 (16)	0.74402 (17)	0.0349 (5)	
C2B	0.8416 (2)	1.15504 (18)	0.76542 (17)	0.0374 (5)	
H2B1	0.829478	1.087239	0.825002	0.045*	
H2B2	0.864727	1.212116	0.783296	0.045*	
C3B	0.94100 (19)	1.15475 (17)	0.66314 (16)	0.0346 (5)	
H3B1	0.993853	1.099103	0.678047	0.042*	
H3B2	0.994039	1.223394	0.620961	0.042*	
C4B	0.86699 (18)	1.13305 (16)	0.60480 (15)	0.0280 (4)	
C5B	0.85239 (19)	1.01823 (15)	0.61969 (15)	0.0282 (4)	
H5B	0.935272	1.000615	0.581203	0.034*	
C6B	0.7592 (2)	0.98840 (17)	0.58105 (16)	0.0351 (5)	
H6B1	0.729247	1.051475	0.542514	0.042*	
H6B2	0.796855	0.950216	0.533855	0.042*	
C7B	0.65457 (19)	0.91825 (16)	0.68284 (16)	0.0310 (5)	
H7B1	0.591918	0.959725	0.712633	0.037*	
H7B2	0.613531	0.864893	0.671045	0.037*	
C8B	0.72199 (17)	0.86788 (15)	0.75470 (15)	0.0256 (4)	
H8B	0.767863	0.813131	0.732103	0.031*	
C9B	0.64364 (17)	0.81890 (15)	0.87288 (15)	0.0248 (4)	
C10B	0.53397 (17)	0.74261 (15)	0.89629 (16)	0.0284 (4)	
H10A	0.457917	0.777043	0.903113	0.034*	
H10B	0.552599	0.714548	0.839949	0.034*	
C11B	0.52016 (17)	0.65634 (16)	1.00148 (16)	0.0282 (4)	
H11C	0.477615	0.589592	1.012303	0.034*	
H11D	0.474249	0.676778	1.062090	0.034*	
C12B	0.65513 (17)	0.64795 (14)	0.98652 (15)	0.0244 (4)	
C13B	0.67927 (17)	0.60497 (16)	1.08886 (15)	0.0287 (4)	
C14B	0.61423 (19)	0.49183 (16)	1.15659 (17)	0.0346 (5)	
H14A	0.592341	0.475375	1.232892	0.042*	
H14B	0.537857	0.480973	1.146110	0.042*	
C15B	0.7095 (2)	0.42414 (17)	1.11669 (18)	0.0393 (5)	
H15A	0.733957	0.377801	1.173765	0.047*	
H15B	0.676818	0.380541	1.089258	0.047*	
C16B	0.81841 (19)	0.50423 (17)	1.02812 (17)	0.0338 (5)	
H16B	0.898251	0.475607	1.024940	0.041*	
C17B	0.81419 (17)	0.54378 (17)	0.91854 (17)	0.0313 (5)	
C18B	0.8173 (2)	0.45619 (19)	0.8788 (2)	0.0437 (6)	
H18D	0.815124	0.484935	0.808142	0.065*	
H18E	0.745160	0.402990	0.927795	0.065*	
H18F	0.893443	0.424105	0.874821	0.065*	
C19B	0.92101 (19)	0.62996 (19)	0.83865 (18)	0.0417 (5)	
H19D	0.918182	0.687035	0.863745	0.063*	
H19E	0.913827	0.656955	0.769899	0.063*	
H19F	0.999839	0.601432	0.830839	0.063*	
C20B	0.6535 (2)	0.67817 (18)	1.14691 (17)	0.0385 (5)	
H20D	0.707216	0.745088	1.100893	0.058*	
H20E	0.670064	0.646793	1.211257	0.058*	
H20F	0.566500	0.690312	1.166389	0.058*	
C21B	0.60291 (19)	0.89926 (16)	0.92287 (16)	0.0320 (5)	
H21A	0.675150	0.933495	0.922613	0.048*	
H21B	0.542534	0.864114	0.996128	0.048*	
H21C	0.564753	0.952034	0.882138	0.048*	
C22B	0.9201 (2)	1.20377 (17)	0.48758 (16)	0.0391 (5)	
H22A	0.931551	1.276899	0.479708	0.059*	
H22B	0.862717	1.196301	0.455859	0.059*	
H22C	1.000195	1.183691	0.451622	0.059*	
O1B	0.74377 (13)	1.16057 (11)	0.65307 (11)	0.0326 (3)	
O2B	0.81091 (12)	0.95312 (10)	0.73040 (10)	0.0287 (3)	
O3B	0.71721 (11)	0.75179 (10)	0.92470 (10)	0.0261 (3)	
O4B	0.69548 (11)	0.58174 (10)	0.92806 (10)	0.0277 (3)	
O5B	0.80931 (12)	0.59184 (11)	1.05972 (11)	0.0331 (3)	
O6B	0.63093 (15)	1.19412 (13)	0.79565 (13)	0.0491 (4)	

### Atomic displacement parameters (Å^2^)

**Table:**

	*U*^11^	*U*^22^	*U*^33^	*U*^12^	*U*^13^	*U*^23^
O1A	0.046 (2)	0.0343 (18)	0.0352 (13)	0.0167 (16)	−0.0199 (15)	−0.0130 (11)
O6A	0.090 (3)	0.0528 (17)	0.0419 (14)	0.0319 (18)	−0.0168 (16)	−0.0245 (12)
C1A	0.048 (3)	0.0219 (16)	0.0321 (16)	0.007 (2)	−0.013 (2)	−0.0048 (12)
C2A	0.036 (2)	0.0289 (19)	0.0316 (15)	−0.0011 (15)	−0.0157 (15)	−0.0078 (13)
C3A	0.025 (2)	0.0326 (19)	0.0345 (16)	−0.0003 (14)	−0.0110 (15)	−0.0097 (13)
C4A	0.027 (2)	0.0172 (17)	0.024 (2)	−0.0065 (16)	−0.0042 (18)	−0.0038 (15)
C5A	0.044 (2)	0.023 (2)	0.0247 (19)	−0.0046 (17)	−0.0201 (13)	−0.0057 (12)
C22A	0.056 (4)	0.0252 (18)	0.021 (2)	−0.0007 (17)	−0.008 (2)	0.0015 (18)
C6A	0.073 (3)	0.043 (3)	0.054 (2)	−0.009 (2)	−0.045 (2)	−0.0011 (18)
C7A	0.0403 (13)	0.0460 (15)	0.0430 (13)	0.0095 (11)	−0.0244 (11)	−0.0133 (11)
O1AA	0.061 (9)	0.022 (5)	0.040 (6)	0.007 (7)	−0.021 (7)	−0.011 (4)
O6AA	0.124 (14)	0.048 (8)	0.044 (8)	−0.005 (10)	0.005 (10)	−0.013 (6)
C1AA	0.061 (10)	0.019 (6)	0.042 (8)	−0.006 (9)	−0.005 (9)	−0.004 (6)
C2AA	0.048 (10)	0.030 (8)	0.028 (6)	−0.002 (7)	−0.013 (8)	−0.012 (6)
C3AA	0.034 (9)	0.043 (9)	0.052 (8)	−0.007 (7)	−0.009 (7)	−0.001 (7)
C4AA	0.014 (8)	0.045 (8)	0.014 (6)	−0.014 (6)	−0.008 (6)	0.006 (6)
C5AA	0.049 (5)	0.017 (4)	0.023 (5)	−0.011 (4)	−0.019 (5)	−0.005 (4)
C22C	0.055 (15)	0.058 (11)	0.033 (11)	−0.011 (10)	0.007 (10)	−0.030 (9)
C6AA	0.065 (8)	0.027 (8)	0.058 (8)	0.000 (7)	−0.057 (6)	−0.004 (6)
C7AA	0.0403 (13)	0.0460 (15)	0.0430 (13)	0.0095 (11)	−0.0244 (11)	−0.0133 (11)
C8A	0.0289 (10)	0.0235 (11)	0.0325 (11)	0.0022 (8)	−0.0153 (9)	−0.0110 (9)
C9A	0.0256 (10)	0.0234 (11)	0.0325 (11)	0.0032 (8)	−0.0128 (8)	−0.0110 (9)
C10A	0.0228 (10)	0.0311 (12)	0.0406 (12)	0.0009 (8)	−0.0115 (9)	−0.0137 (10)
C11A	0.0266 (10)	0.0283 (12)	0.0356 (11)	−0.0016 (8)	−0.0060 (9)	−0.0134 (10)
C12A	0.0255 (10)	0.0207 (10)	0.0269 (10)	−0.0023 (8)	−0.0074 (8)	−0.0082 (8)
C13A	0.0319 (11)	0.0233 (11)	0.0250 (10)	−0.0026 (8)	−0.0071 (8)	−0.0071 (9)
C14A	0.0453 (13)	0.0264 (12)	0.0269 (11)	−0.0020 (9)	−0.0114 (9)	−0.0034 (9)
C15A	0.0491 (13)	0.0230 (12)	0.0400 (13)	0.0029 (10)	−0.0215 (11)	−0.0068 (10)
C16A	0.0303 (11)	0.0301 (12)	0.0393 (12)	0.0064 (9)	−0.0166 (9)	−0.0162 (10)
C17A	0.0213 (9)	0.0309 (12)	0.0324 (11)	0.0047 (8)	−0.0102 (8)	−0.0145 (9)
C18A	0.0343 (11)	0.0381 (13)	0.0434 (13)	0.0072 (10)	−0.0147 (10)	−0.0230 (11)
C19A	0.0279 (11)	0.0404 (13)	0.0306 (11)	−0.0011 (9)	−0.0057 (9)	−0.0135 (10)
C20A	0.0514 (14)	0.0330 (13)	0.0303 (11)	−0.0001 (10)	−0.0157 (10)	−0.0116 (10)
C21A	0.0445 (13)	0.0301 (12)	0.0346 (12)	0.0052 (10)	−0.0125 (10)	−0.0154 (10)
O2A	0.0342 (8)	0.0242 (8)	0.0280 (7)	−0.0050 (6)	−0.0156 (6)	−0.0019 (6)
O3A	0.0251 (7)	0.0188 (7)	0.0301 (7)	−0.0024 (5)	−0.0119 (6)	−0.0033 (6)
O4A	0.0236 (7)	0.0278 (8)	0.0302 (7)	0.0013 (6)	−0.0100 (6)	−0.0139 (6)
O5A	0.0316 (7)	0.0303 (8)	0.0330 (8)	0.0007 (6)	−0.0162 (6)	−0.0117 (7)
C1B	0.0379 (12)	0.0254 (12)	0.0343 (12)	−0.0073 (9)	−0.0071 (10)	−0.0105 (10)
C2B	0.0437 (13)	0.0363 (13)	0.0300 (11)	−0.0083 (10)	−0.0117 (10)	−0.0124 (10)
C3B	0.0346 (11)	0.0324 (12)	0.0331 (11)	−0.0071 (9)	−0.0128 (9)	−0.0084 (10)
C4B	0.0301 (10)	0.0255 (11)	0.0253 (10)	0.0005 (8)	−0.0092 (8)	−0.0082 (9)
C5B	0.0346 (11)	0.0257 (11)	0.0217 (10)	0.0021 (8)	−0.0108 (8)	−0.0066 (9)
C6B	0.0510 (13)	0.0282 (12)	0.0310 (11)	0.0020 (10)	−0.0216 (10)	−0.0109 (10)
C7B	0.0385 (12)	0.0283 (12)	0.0344 (11)	0.0045 (9)	−0.0195 (9)	−0.0154 (9)
C8B	0.0286 (10)	0.0200 (10)	0.0295 (10)	0.0016 (8)	−0.0118 (8)	−0.0108 (8)
C9B	0.0251 (10)	0.0209 (10)	0.0287 (10)	0.0024 (8)	−0.0121 (8)	−0.0082 (8)
C10B	0.0253 (10)	0.0252 (11)	0.0368 (11)	0.0029 (8)	−0.0152 (9)	−0.0111 (9)
C11B	0.0237 (10)	0.0240 (11)	0.0347 (11)	−0.0002 (8)	−0.0096 (8)	−0.0107 (9)
C12B	0.0231 (9)	0.0180 (10)	0.0308 (10)	−0.0012 (8)	−0.0094 (8)	−0.0089 (8)
C13B	0.0261 (10)	0.0266 (11)	0.0277 (10)	−0.0021 (8)	−0.0074 (8)	−0.0072 (9)
C14B	0.0358 (12)	0.0279 (12)	0.0327 (11)	−0.0041 (9)	−0.0131 (9)	−0.0035 (9)
C15B	0.0442 (13)	0.0288 (12)	0.0427 (13)	0.0036 (10)	−0.0209 (11)	−0.0075 (10)
C16B	0.0292 (11)	0.0322 (12)	0.0458 (13)	0.0085 (9)	−0.0193 (10)	−0.0167 (10)
C17B	0.0227 (10)	0.0329 (12)	0.0402 (12)	0.0067 (8)	−0.0111 (9)	−0.0175 (10)
C18B	0.0416 (13)	0.0436 (15)	0.0555 (15)	0.0153 (11)	−0.0199 (11)	−0.0295 (13)
C19B	0.0296 (11)	0.0460 (15)	0.0436 (13)	0.0045 (10)	−0.0061 (10)	−0.0195 (11)
C20B	0.0479 (13)	0.0375 (13)	0.0331 (12)	0.0008 (10)	−0.0182 (10)	−0.0143 (10)
C21B	0.0378 (12)	0.0261 (11)	0.0313 (11)	0.0028 (9)	−0.0119 (9)	−0.0117 (9)
C22B	0.0514 (14)	0.0291 (12)	0.0288 (11)	−0.0001 (10)	−0.0135 (10)	−0.0044 (10)
O1B	0.0364 (8)	0.0287 (8)	0.0346 (8)	0.0047 (6)	−0.0143 (6)	−0.0138 (7)
O2B	0.0339 (7)	0.0242 (8)	0.0252 (7)	−0.0052 (6)	−0.0138 (6)	−0.0036 (6)
O3B	0.0260 (7)	0.0197 (7)	0.0300 (7)	−0.0022 (5)	−0.0138 (6)	−0.0035 (6)
O4B	0.0261 (7)	0.0277 (8)	0.0331 (8)	0.0047 (6)	−0.0127 (6)	−0.0151 (6)
O5B	0.0293 (7)	0.0328 (8)	0.0418 (8)	0.0013 (6)	−0.0182 (6)	−0.0148 (7)
O6B	0.0419 (9)	0.0495 (11)	0.0485 (10)	0.0008 (8)	−0.0032 (8)	−0.0260 (9)

### Geometric parameters (Å, º)

**Table:**

O1A—C1A	1.353 (6)	C18A—H18A	0.9800
O1A—C4A	1.464 (5)	C18A—H18B	0.9800
O6A—C1A	1.200 (6)	C18A—H18C	0.9800
C1A—C2A	1.493 (5)	C19A—H19A	0.9800
C2A—C3A	1.511 (5)	C19A—H19B	0.9800
C2A—H2A1	0.9900	C19A—H19C	0.9800
C2A—H2A2	0.9900	C20A—H20A	0.9800
C3A—C4A	1.533 (6)	C20A—H20B	0.9800
C3A—H3A1	0.9900	C20A—H20C	0.9800
C3A—H3A2	0.9900	C21A—H21D	0.9800
C4A—C5A	1.514 (5)	C21A—H21E	0.9800
C4A—C22A	1.525 (5)	C21A—H21F	0.9800
C5A—O2A	1.412 (9)	C1B—O6B	1.202 (3)
C5A—C6A	1.550 (6)	C1B—O1B	1.348 (2)
C5A—H5A	1.0000	C1B—C2B	1.491 (3)
C22A—H22D	0.9800	C2B—C3B	1.519 (3)
C22A—H22E	0.9800	C2B—H2B1	0.9900
C22A—H22F	0.9800	C2B—H2B2	0.9900
C6A—C7A	1.511 (5)	C3B—C4B	1.533 (3)
C6A—H6A1	0.9900	C3B—H3B1	0.9900
C6A—H6A2	0.9900	C3B—H3B2	0.9900
C7A—C8A	1.517 (3)	C4B—O1B	1.462 (2)
C7A—H7A1	0.9900	C4B—C22B	1.521 (3)
C7A—H7A2	0.9900	C4B—C5B	1.524 (3)
O1AA—C1AA	1.36 (4)	C5B—O2B	1.435 (2)
O1AA—C4AA	1.418 (19)	C5B—C6B	1.535 (3)
O6AA—C1AA	1.17 (3)	C5B—H5B	1.0000
C1AA—C2AA	1.476 (16)	C6B—C7B	1.518 (3)
C2AA—C3AA	1.494 (18)	C6B—H6B1	0.9900
C2AA—H2A3	0.9900	C6B—H6B2	0.9900
C2AA—H2A4	0.9900	C7B—C8B	1.517 (3)
C3AA—C4AA	1.533 (18)	C7B—H7B1	0.9900
C3AA—H3A3	0.9900	C7B—H7B2	0.9900
C3AA—H3A4	0.9900	C8B—O2B	1.441 (2)
C4AA—C5AA	1.499 (17)	C8B—C9B	1.516 (3)
C4AA—C22C	1.520 (17)	C8B—H8B	1.0000
C5AA—O2A	1.53 (5)	C9B—O3B	1.460 (2)
C5AA—C6AA	1.531 (18)	C9B—C21B	1.518 (3)
C5AA—H5AA	1.0000	C9B—C10B	1.535 (3)
C22C—H22G	0.9800	C10B—C11B	1.524 (3)
C22C—H22H	0.9800	C10B—H10A	0.9900
C22C—H22I	0.9800	C10B—H10B	0.9900
C6AA—C7AA	1.476 (16)	C11B—C12B	1.524 (3)
C6AA—H6A3	0.9900	C11B—H11C	0.9900
C6AA—H6A4	0.9900	C11B—H11D	0.9900
C7AA—C8A	1.517 (3)	C12B—O3B	1.419 (2)
C7AA—H7A3	0.9900	C12B—O4B	1.430 (2)
C7AA—H7A4	0.9900	C12B—C13B	1.539 (3)
C8A—O2A	1.436 (2)	C13B—O5B	1.449 (2)
C8A—C9A	1.515 (3)	C13B—C20B	1.505 (3)
C8A—H8A	1.0000	C13B—C14B	1.538 (3)
C9A—O3A	1.456 (2)	C14B—C15B	1.524 (3)
C9A—C21A	1.525 (3)	C14B—H14A	0.9900
C9A—C10A	1.535 (3)	C14B—H14B	0.9900
C10A—C11A	1.524 (3)	C15B—C16B	1.523 (3)
C10A—H10C	0.9900	C15B—H15A	0.9900
C10A—H10D	0.9900	C15B—H15B	0.9900
C11A—C12A	1.522 (3)	C16B—O5B	1.434 (2)
C11A—H11A	0.9900	C16B—C17B	1.525 (3)
C11A—H11B	0.9900	C16B—H16B	1.0000
C12A—O3A	1.415 (2)	C17B—O4B	1.452 (2)
C12A—O4A	1.433 (2)	C17B—C18B	1.518 (3)
C12A—C13A	1.541 (3)	C17B—C19B	1.521 (3)
C13A—O5A	1.455 (2)	C18B—H18D	0.9800
C13A—C20A	1.511 (3)	C18B—H18E	0.9800
C13A—C14A	1.538 (3)	C18B—H18F	0.9800
C14A—C15A	1.535 (3)	C19B—H19D	0.9800
C14A—H14C	0.9900	C19B—H19E	0.9800
C14A—H14D	0.9900	C19B—H19F	0.9800
C15A—C16A	1.522 (3)	C20B—H20D	0.9800
C15A—H15C	0.9900	C20B—H20E	0.9800
C15A—H15D	0.9900	C20B—H20F	0.9800
C16A—O5A	1.438 (2)	C21B—H21A	0.9800
C16A—C17A	1.524 (3)	C21B—H21B	0.9800
C16A—H16A	1.0000	C21B—H21C	0.9800
C17A—O4A	1.448 (2)	C22B—H22A	0.9800
C17A—C18A	1.521 (3)	C22B—H22B	0.9800
C17A—C19A	1.523 (3)	C22B—H22C	0.9800
			
C1A—O1A—C4A	111.5 (4)	H18B—C18A—H18C	109.5
O6A—C1A—O1A	120.9 (4)	C17A—C19A—H19A	109.5
O6A—C1A—C2A	129.0 (4)	C17A—C19A—H19B	109.5
O1A—C1A—C2A	110.1 (4)	H19A—C19A—H19B	109.5
C1A—C2A—C3A	105.2 (3)	C17A—C19A—H19C	109.5
C1A—C2A—H2A1	110.7	H19A—C19A—H19C	109.5
C3A—C2A—H2A1	110.7	H19B—C19A—H19C	109.5
C1A—C2A—H2A2	110.7	C13A—C20A—H20A	109.5
C3A—C2A—H2A2	110.7	C13A—C20A—H20B	109.5
H2A1—C2A—H2A2	108.8	H20A—C20A—H20B	109.5
C2A—C3A—C4A	104.4 (3)	C13A—C20A—H20C	109.5
C2A—C3A—H3A1	110.9	H20A—C20A—H20C	109.5
C4A—C3A—H3A1	110.9	H20B—C20A—H20C	109.5
C2A—C3A—H3A2	110.9	C9A—C21A—H21D	109.5
C4A—C3A—H3A2	110.9	C9A—C21A—H21E	109.5
H3A1—C3A—H3A2	108.9	H21D—C21A—H21E	109.5
O1A—C4A—C5A	107.5 (4)	C9A—C21A—H21F	109.5
O1A—C4A—C22A	109.2 (4)	H21D—C21A—H21F	109.5
C5A—C4A—C22A	111.4 (6)	H21E—C21A—H21F	109.5
O1A—C4A—C3A	104.8 (3)	C5A—O2A—C8A	110.0 (2)
C5A—C4A—C3A	108.9 (4)	C8A—O2A—C5AA	98.7 (10)
C22A—C4A—C3A	114.6 (5)	C12A—O3A—C9A	111.45 (14)
O2A—C5A—C4A	111.9 (5)	C12A—O4A—C17A	117.81 (14)
O2A—C5A—C6A	105.1 (5)	C16A—O5A—C13A	103.34 (14)
C4A—C5A—C6A	119.6 (5)	O6B—C1B—O1B	121.6 (2)
O2A—C5A—H5A	106.5	O6B—C1B—C2B	128.4 (2)
C4A—C5A—H5A	106.5	O1B—C1B—C2B	110.03 (18)
C6A—C5A—H5A	106.5	C1B—C2B—C3B	105.16 (17)
C4A—C22A—H22D	109.5	C1B—C2B—H2B1	110.7
C4A—C22A—H22E	109.5	C3B—C2B—H2B1	110.7
H22D—C22A—H22E	109.5	C1B—C2B—H2B2	110.7
C4A—C22A—H22F	109.5	C3B—C2B—H2B2	110.7
H22D—C22A—H22F	109.5	H2B1—C2B—H2B2	108.8
H22E—C22A—H22F	109.5	C2B—C3B—C4B	103.78 (16)
C7A—C6A—C5A	105.5 (5)	C2B—C3B—H3B1	111.0
C7A—C6A—H6A1	110.6	C4B—C3B—H3B1	111.0
C5A—C6A—H6A1	110.6	C2B—C3B—H3B2	111.0
C7A—C6A—H6A2	110.6	C4B—C3B—H3B2	111.0
C5A—C6A—H6A2	110.6	H3B1—C3B—H3B2	109.0
H6A1—C6A—H6A2	108.8	O1B—C4B—C22B	107.36 (16)
C6A—C7A—C8A	102.6 (3)	O1B—C4B—C5B	108.01 (15)
C6A—C7A—H7A1	111.3	C22B—C4B—C5B	110.79 (16)
C8A—C7A—H7A1	111.3	O1B—C4B—C3B	104.90 (15)
C6A—C7A—H7A2	111.3	C22B—C4B—C3B	111.96 (17)
C8A—C7A—H7A2	111.3	C5B—C4B—C3B	113.39 (17)
H7A1—C7A—H7A2	109.2	O2B—C5B—C4B	109.47 (15)
C1AA—O1AA—C4AA	111 (2)	O2B—C5B—C6B	106.46 (16)
O6AA—C1AA—O1AA	120 (2)	C4B—C5B—C6B	115.39 (17)
O6AA—C1AA—C2AA	131 (3)	O2B—C5B—H5B	108.4
O1AA—C1AA—C2AA	109 (2)	C4B—C5B—H5B	108.4
C1AA—C2AA—C3AA	105.3 (19)	C6B—C5B—H5B	108.4
C1AA—C2AA—H2A3	110.7	C7B—C6B—C5B	103.64 (16)
C3AA—C2AA—H2A3	110.7	C7B—C6B—H6B1	111.0
C1AA—C2AA—H2A4	110.7	C5B—C6B—H6B1	111.0
C3AA—C2AA—H2A4	110.7	C7B—C6B—H6B2	111.0
H2A3—C2AA—H2A4	108.8	C5B—C6B—H6B2	111.0
C2AA—C3AA—C4AA	102.4 (17)	H6B1—C6B—H6B2	109.0
C2AA—C3AA—H3A3	111.3	C8B—C7B—C6B	102.36 (16)
C4AA—C3AA—H3A3	111.3	C8B—C7B—H7B1	111.3
C2AA—C3AA—H3A4	111.3	C6B—C7B—H7B1	111.3
C4AA—C3AA—H3A4	111.3	C8B—C7B—H7B2	111.3
H3A3—C3AA—H3A4	109.2	C6B—C7B—H7B2	111.3
O1AA—C4AA—C5AA	113 (2)	H7B1—C7B—H7B2	109.2
O1AA—C4AA—C22C	95 (2)	O2B—C8B—C9B	110.87 (14)
C5AA—C4AA—C22C	112 (3)	O2B—C8B—C7B	102.99 (15)
O1AA—C4AA—C3AA	104.2 (18)	C9B—C8B—C7B	116.84 (16)
C5AA—C4AA—C3AA	127 (2)	O2B—C8B—H8B	108.6
C22C—C4AA—C3AA	100 (3)	C9B—C8B—H8B	108.6
C4AA—C5AA—O2A	92 (2)	C7B—C8B—H8B	108.6
C4AA—C5AA—C6AA	95 (2)	O3B—C9B—C8B	108.28 (15)
O2A—C5AA—C6AA	106 (3)	O3B—C9B—C21B	107.89 (15)
C4AA—C5AA—H5AA	119.5	C8B—C9B—C21B	113.21 (16)
O2A—C5AA—H5AA	119.5	O3B—C9B—C10B	103.86 (15)
C6AA—C5AA—H5AA	119.5	C8B—C9B—C10B	110.72 (15)
C4AA—C22C—H22G	109.5	C21B—C9B—C10B	112.34 (16)
C4AA—C22C—H22H	109.5	C11B—C10B—C9B	102.84 (15)
H22G—C22C—H22H	109.5	C11B—C10B—H10A	111.2
C4AA—C22C—H22I	109.5	C9B—C10B—H10A	111.2
H22G—C22C—H22I	109.5	C11B—C10B—H10B	111.2
H22H—C22C—H22I	109.5	C9B—C10B—H10B	111.2
C7AA—C6AA—C5AA	96.0 (19)	H10A—C10B—H10B	109.1
C7AA—C6AA—H6A3	112.5	C10B—C11B—C12B	101.56 (15)
C5AA—C6AA—H6A3	112.5	C10B—C11B—H11C	111.5
C7AA—C6AA—H6A4	112.5	C12B—C11B—H11C	111.5
C5AA—C6AA—H6A4	112.5	C10B—C11B—H11D	111.5
H6A3—C6AA—H6A4	110.1	C12B—C11B—H11D	111.5
C6AA—C7AA—C8A	112.1 (9)	H11C—C11B—H11D	109.3
C6AA—C7AA—H7A3	109.2	O3B—C12B—O4B	110.98 (14)
C8A—C7AA—H7A3	109.2	O3B—C12B—C11B	105.08 (15)
C6AA—C7AA—H7A4	109.2	O4B—C12B—C11B	104.98 (14)
C8A—C7AA—H7A4	109.2	O3B—C12B—C13B	109.10 (14)
H7A3—C7AA—H7A4	107.9	O4B—C12B—C13B	110.59 (15)
O2A—C8A—C9A	109.92 (15)	C11B—C12B—C13B	115.95 (16)
O2A—C8A—C7A	103.93 (16)	O5B—C13B—C20B	107.19 (16)
C9A—C8A—C7A	117.02 (17)	O5B—C13B—C14B	102.72 (16)
O2A—C8A—C7AA	103.93 (16)	C20B—C13B—C14B	115.09 (17)
C9A—C8A—C7AA	117.02 (17)	O5B—C13B—C12B	107.96 (15)
O2A—C8A—H8A	108.5	C20B—C13B—C12B	113.03 (17)
C9A—C8A—H8A	108.5	C14B—C13B—C12B	110.06 (16)
C7A—C8A—H8A	108.5	C15B—C14B—C13B	104.27 (16)
O3A—C9A—C8A	106.95 (15)	C15B—C14B—H14A	110.9
O3A—C9A—C21A	107.88 (15)	C13B—C14B—H14A	110.9
C8A—C9A—C21A	113.05 (17)	C15B—C14B—H14B	110.9
O3A—C9A—C10A	104.33 (15)	C13B—C14B—H14B	110.9
C8A—C9A—C10A	112.00 (16)	H14A—C14B—H14B	108.9
C21A—C9A—C10A	112.03 (17)	C16B—C15B—C14B	103.42 (17)
C11A—C10A—C9A	102.66 (16)	C16B—C15B—H15A	111.1
C11A—C10A—H10C	111.2	C14B—C15B—H15A	111.1
C9A—C10A—H10C	111.2	C16B—C15B—H15B	111.1
C11A—C10A—H10D	111.2	C14B—C15B—H15B	111.1
C9A—C10A—H10D	111.2	H15A—C15B—H15B	109.0
H10C—C10A—H10D	109.1	O5B—C16B—C15B	103.74 (17)
C12A—C11A—C10A	101.66 (16)	O5B—C16B—C17B	107.34 (17)
C12A—C11A—H11A	111.4	C15B—C16B—C17B	115.43 (17)
C10A—C11A—H11A	111.4	O5B—C16B—H16B	110.0
C12A—C11A—H11B	111.4	C15B—C16B—H16B	110.0
C10A—C11A—H11B	111.4	C17B—C16B—H16B	110.0
H11A—C11A—H11B	109.3	O4B—C17B—C18B	105.42 (16)
O3A—C12A—O4A	112.13 (15)	O4B—C17B—C19B	111.07 (17)
O3A—C12A—C11A	104.77 (15)	C18B—C17B—C19B	109.49 (18)
O4A—C12A—C11A	104.51 (15)	O4B—C17B—C16B	107.82 (15)
O3A—C12A—C13A	107.64 (14)	C18B—C17B—C16B	112.08 (18)
O4A—C12A—C13A	109.83 (15)	C19B—C17B—C16B	110.85 (17)
C11A—C12A—C13A	117.95 (16)	C17B—C18B—H18D	109.5
O5A—C13A—C20A	107.20 (16)	C17B—C18B—H18E	109.5
O5A—C13A—C14A	103.47 (16)	H18D—C18B—H18E	109.5
C20A—C13A—C14A	115.17 (17)	C17B—C18B—H18F	109.5
O5A—C13A—C12A	106.63 (15)	H18D—C18B—H18F	109.5
C20A—C13A—C12A	113.08 (17)	H18E—C18B—H18F	109.5
C14A—C13A—C12A	110.42 (16)	C17B—C19B—H19D	109.5
C15A—C14A—C13A	103.99 (16)	C17B—C19B—H19E	109.5
C15A—C14A—H14C	111.0	H19D—C19B—H19E	109.5
C13A—C14A—H14C	111.0	C17B—C19B—H19F	109.5
C15A—C14A—H14D	111.0	H19D—C19B—H19F	109.5
C13A—C14A—H14D	111.0	H19E—C19B—H19F	109.5
H14C—C14A—H14D	109.0	C13B—C20B—H20D	109.5
C16A—C15A—C14A	103.55 (17)	C13B—C20B—H20E	109.5
C16A—C15A—H15C	111.1	H20D—C20B—H20E	109.5
C14A—C15A—H15C	111.1	C13B—C20B—H20F	109.5
C16A—C15A—H15D	111.1	H20D—C20B—H20F	109.5
C14A—C15A—H15D	111.1	H20E—C20B—H20F	109.5
H15C—C15A—H15D	109.0	C9B—C21B—H21A	109.5
O5A—C16A—C15A	102.84 (16)	C9B—C21B—H21B	109.5
O5A—C16A—C17A	107.85 (16)	H21A—C21B—H21B	109.5
C15A—C16A—C17A	115.43 (17)	C9B—C21B—H21C	109.5
O5A—C16A—H16A	110.1	H21A—C21B—H21C	109.5
C15A—C16A—H16A	110.1	H21B—C21B—H21C	109.5
C17A—C16A—H16A	110.1	C4B—C22B—H22A	109.5
O4A—C17A—C18A	106.01 (15)	C4B—C22B—H22B	109.5
O4A—C17A—C19A	110.16 (16)	H22A—C22B—H22B	109.5
C18A—C17A—C19A	109.42 (17)	C4B—C22B—H22C	109.5
O4A—C17A—C16A	108.77 (15)	H22A—C22B—H22C	109.5
C18A—C17A—C16A	111.31 (17)	H22B—C22B—H22C	109.5
C19A—C17A—C16A	111.04 (16)	C1B—O1B—C4B	111.83 (16)
C17A—C18A—H18A	109.5	C5B—O2B—C8B	108.66 (14)
C17A—C18A—H18B	109.5	C12B—O3B—C9B	111.50 (13)
H18A—C18A—H18B	109.5	C12B—O4B—C17B	117.36 (14)
C17A—C18A—H18C	109.5	C16B—O5B—C13B	103.05 (14)
H18A—C18A—H18C	109.5		
			
C4A—O1A—C1A—O6A	−178.2 (4)	C8A—C9A—O3A—C12A	−123.21 (15)
C4A—O1A—C1A—C2A	2.4 (5)	C21A—C9A—O3A—C12A	114.90 (17)
O6A—C1A—C2A—C3A	−168.8 (5)	C10A—C9A—O3A—C12A	−4.39 (19)
O1A—C1A—C2A—C3A	10.5 (5)	O3A—C12A—O4A—C17A	75.1 (2)
C1A—C2A—C3A—C4A	−18.2 (4)	C11A—C12A—O4A—C17A	−171.95 (15)
C1A—O1A—C4A—C5A	101.7 (5)	C13A—C12A—O4A—C17A	−44.5 (2)
C1A—O1A—C4A—C22A	−137.3 (6)	C18A—C17A—O4A—C12A	164.31 (16)
C1A—O1A—C4A—C3A	−14.0 (5)	C19A—C17A—O4A—C12A	−77.4 (2)
C2A—C3A—C4A—O1A	19.5 (4)	C16A—C17A—O4A—C12A	44.5 (2)
C2A—C3A—C4A—C5A	−95.3 (5)	C15A—C16A—O5A—C13A	−48.12 (18)
C2A—C3A—C4A—C22A	139.2 (5)	C17A—C16A—O5A—C13A	74.30 (18)
O1A—C4A—C5A—O2A	−65.7 (5)	C20A—C13A—O5A—C16A	165.85 (16)
C22A—C4A—C5A—O2A	174.7 (4)	C14A—C13A—O5A—C16A	43.71 (18)
C3A—C4A—C5A—O2A	47.3 (5)	C12A—C13A—O5A—C16A	−72.77 (17)
O1A—C4A—C5A—C6A	57.8 (8)	O6B—C1B—C2B—C3B	−167.3 (2)
C22A—C4A—C5A—C6A	−61.8 (8)	O1B—C1B—C2B—C3B	12.1 (2)
C3A—C4A—C5A—C6A	170.8 (6)	C1B—C2B—C3B—C4B	−19.4 (2)
O2A—C5A—C6A—C7A	3.7 (6)	C2B—C3B—C4B—O1B	20.0 (2)
C4A—C5A—C6A—C7A	−122.9 (6)	C2B—C3B—C4B—C22B	136.08 (19)
C5A—C6A—C7A—C8A	−23.3 (5)	C2B—C3B—C4B—C5B	−97.7 (2)
C4AA—O1AA—C1AA—O6AA	−167 (2)	O1B—C4B—C5B—O2B	−66.91 (19)
C4AA—O1AA—C1AA—C2AA	10 (3)	C22B—C4B—C5B—O2B	175.77 (16)
O6AA—C1AA—C2AA—C3AA	−174 (3)	C3B—C4B—C5B—O2B	48.9 (2)
O1AA—C1AA—C2AA—C3AA	9 (3)	O1B—C4B—C5B—C6B	53.1 (2)
C1AA—C2AA—C3AA—C4AA	−23 (3)	C22B—C4B—C5B—C6B	−64.2 (2)
C1AA—O1AA—C4AA—C5AA	118 (3)	C3B—C4B—C5B—C6B	168.92 (16)
C1AA—O1AA—C4AA—C22C	−125 (3)	O2B—C5B—C6B—C7B	10.0 (2)
C1AA—O1AA—C4AA—C3AA	−24 (3)	C4B—C5B—C6B—C7B	−111.69 (19)
C2AA—C3AA—C4AA—O1AA	28 (3)	C5B—C6B—C7B—C8B	−30.1 (2)
C2AA—C3AA—C4AA—C5AA	−106 (3)	C6B—C7B—C8B—O2B	39.82 (18)
C2AA—C3AA—C4AA—C22C	126 (2)	C6B—C7B—C8B—C9B	161.60 (16)
O1AA—C4AA—C5AA—O2A	−83 (2)	O2B—C8B—C9B—O3B	−77.34 (18)
C22C—C4AA—C5AA—O2A	170 (2)	C7B—C8B—C9B—O3B	165.11 (15)
C3AA—C4AA—C5AA—O2A	48 (3)	O2B—C8B—C9B—C21B	42.2 (2)
O1AA—C4AA—C5AA—C6AA	23 (4)	C7B—C8B—C9B—C21B	−75.3 (2)
C22C—C4AA—C5AA—C6AA	−83 (3)	O2B—C8B—C9B—C10B	169.42 (15)
C3AA—C4AA—C5AA—C6AA	154 (2)	C7B—C8B—C9B—C10B	51.9 (2)
C4AA—C5AA—C6AA—C7AA	−133 (2)	O3B—C9B—C10B—C11B	28.65 (18)
O2A—C5AA—C6AA—C7AA	−39 (2)	C8B—C9B—C10B—C11B	144.68 (16)
C5AA—C6AA—C7AA—C8A	16 (2)	C21B—C9B—C10B—C11B	−87.67 (19)
C6A—C7A—C8A—O2A	34.7 (3)	C9B—C10B—C11B—C12B	−38.20 (18)
C6A—C7A—C8A—C9A	156.1 (3)	C10B—C11B—C12B—O3B	34.34 (18)
C6AA—C7AA—C8A—O2A	13.6 (12)	C10B—C11B—C12B—O4B	−82.79 (17)
C6AA—C7AA—C8A—C9A	134.9 (12)	C10B—C11B—C12B—C13B	154.86 (16)
O2A—C8A—C9A—O3A	−67.97 (18)	O3B—C12B—C13B—O5B	−67.88 (18)
C7A—C8A—C9A—O3A	173.84 (16)	O4B—C12B—C13B—O5B	54.45 (19)
C7AA—C8A—C9A—O3A	173.84 (16)	C11B—C12B—C13B—O5B	173.78 (15)
O2A—C8A—C9A—C21A	50.6 (2)	O3B—C12B—C13B—C20B	50.5 (2)
C7A—C8A—C9A—C21A	−67.6 (2)	O4B—C12B—C13B—C20B	172.83 (15)
C7AA—C8A—C9A—C21A	−67.6 (2)	C11B—C12B—C13B—C20B	−67.8 (2)
O2A—C8A—C9A—C10A	178.31 (16)	O3B—C12B—C13B—C14B	−179.27 (15)
C7A—C8A—C9A—C10A	60.1 (2)	O4B—C12B—C13B—C14B	−56.9 (2)
C7AA—C8A—C9A—C10A	60.1 (2)	C11B—C12B—C13B—C14B	62.4 (2)
O3A—C9A—C10A—C11A	26.54 (19)	O5B—C13B—C14B—C15B	−26.0 (2)
C8A—C9A—C10A—C11A	141.86 (16)	C20B—C13B—C14B—C15B	−142.16 (19)
C21A—C9A—C10A—C11A	−89.9 (2)	C12B—C13B—C14B—C15B	88.73 (19)
C9A—C10A—C11A—C12A	−37.60 (19)	C13B—C14B—C15B—C16B	−1.6 (2)
C10A—C11A—C12A—O3A	35.65 (19)	C14B—C15B—C16B—O5B	29.1 (2)
C10A—C11A—C12A—O4A	−82.43 (17)	C14B—C15B—C16B—C17B	−88.0 (2)
C10A—C11A—C12A—C13A	155.29 (16)	O5B—C16B—C17B—O4B	−61.82 (19)
O3A—C12A—C13A—O5A	−65.08 (18)	C15B—C16B—C17B—O4B	53.3 (2)
O4A—C12A—C13A—O5A	57.26 (18)	O5B—C16B—C17B—C18B	−177.39 (16)
C11A—C12A—C13A—O5A	176.79 (15)	C15B—C16B—C17B—C18B	−62.3 (2)
O3A—C12A—C13A—C20A	52.5 (2)	O5B—C16B—C17B—C19B	59.9 (2)
O4A—C12A—C13A—C20A	174.83 (15)	C15B—C16B—C17B—C19B	175.04 (18)
C11A—C12A—C13A—C20A	−65.6 (2)	O6B—C1B—O1B—C4B	−179.49 (19)
O3A—C12A—C13A—C14A	−176.82 (15)	C2B—C1B—O1B—C4B	1.1 (2)
O4A—C12A—C13A—C14A	−54.5 (2)	C22B—C4B—O1B—C1B	−132.90 (17)
C11A—C12A—C13A—C14A	65.0 (2)	C5B—C4B—O1B—C1B	107.58 (17)
O5A—C13A—C14A—C15A	−21.82 (19)	C3B—C4B—O1B—C1B	−13.6 (2)
C20A—C13A—C14A—C15A	−138.47 (19)	C4B—C5B—O2B—C8B	141.03 (16)
C12A—C13A—C14A—C15A	91.95 (19)	C6B—C5B—O2B—C8B	15.7 (2)
C13A—C14A—C15A—C16A	−6.4 (2)	C9B—C8B—O2B—C5B	−160.70 (15)
C14A—C15A—C16A—O5A	32.83 (19)	C7B—C8B—O2B—C5B	−34.98 (19)
C14A—C15A—C16A—C17A	−84.3 (2)	O4B—C12B—O3B—C9B	95.87 (16)
O5A—C16A—C17A—O4A	−58.47 (19)	C11B—C12B—O3B—C9B	−17.09 (19)
C15A—C16A—C17A—O4A	55.8 (2)	C13B—C12B—O3B—C9B	−142.04 (15)
O5A—C16A—C17A—C18A	−174.91 (16)	C8B—C9B—O3B—C12B	−125.12 (15)
C15A—C16A—C17A—C18A	−60.6 (2)	C21B—C9B—O3B—C12B	112.01 (16)
O5A—C16A—C17A—C19A	62.9 (2)	C10B—C9B—O3B—C12B	−7.39 (19)
C15A—C16A—C17A—C19A	177.22 (17)	O3B—C12B—O4B—C17B	78.00 (19)
C4A—C5A—O2A—C8A	150.6 (3)	C11B—C12B—O4B—C17B	−168.98 (15)
C6A—C5A—O2A—C8A	19.3 (5)	C13B—C12B—O4B—C17B	−43.2 (2)
C9A—C8A—O2A—C5A	−160.7 (3)	C18B—C17B—O4B—C12B	166.08 (17)
C7A—C8A—O2A—C5A	−34.7 (3)	C19B—C17B—O4B—C12B	−75.4 (2)
C9A—C8A—O2A—C5AA	−162.3 (13)	C16B—C17B—O4B—C12B	46.2 (2)
C7AA—C8A—O2A—C5AA	−36.3 (13)	C15B—C16B—O5B—C13B	−47.09 (19)
C4AA—C5AA—O2A—C8A	145.5 (14)	C17B—C16B—O5B—C13B	75.55 (18)
C6AA—C5AA—O2A—C8A	49.8 (19)	C20B—C13B—O5B—C16B	167.10 (16)
O4A—C12A—O3A—C9A	92.95 (17)	C14B—C13B—O5B—C16B	45.44 (18)
C11A—C12A—O3A—C9A	−19.82 (19)	C12B—C13B—O5B—C16B	−70.84 (18)
C13A—C12A—O3A—C9A	−146.15 (15)		

### Hydrogen-bond geometry (Å, º)

**Table:**

*D*—H···*A*	*D*—H	H···*A*	*D*···*A*	*D*—H···*A*
C19*A*—H19*A*···O3*A*	0.98	2.39	3.041 (3)	124
C19*B*—H19*D*···O3*B*	0.98	2.46	3.038 (3)	117
C7*A*—H7*A*1···O6*B*^i^	0.99	2.55	3.464 (4)	154

Symmetry code: (i) −*x*+1, −*y*+2, −*z*+1.
